# Unraveling uneven urbanites’ expressed happiness across Chinese cities using geotagged social media data: Key predictors and future climate–happiness associations

**DOI:** 10.1371/journal.pone.0353996

**Published:** 2026-07-16

**Authors:** Yibiao Li, Hui Zhong, Yufei Dong, Lei Lu

**Affiliations:** 1 School of Arts and Design, Lianyungang Technical College, Lianyungang, China; 2 Design Department, Shanghai Normal University, Shanghai, China; 3 Arts Visual Communication Design Department, Shandong University of Arts, Shandong, China; 4 College of Environment and Ecology, Hunan Agricultural University, Changsha, China; East China Normal University, CHINA

## Abstract

Understanding inter-city disparities in urban expressed happiness (EH) and the key predictors for these differences is critical for advancing socially sustainable urban development. However, the key predictors for EH remain poorly understood, and existing studies have largely overlooked the potential association with future climate change. In this study, we analyzed 5,118,772 geotagged Weibo posts from 50 Chinese cities using SnowNLP for sentiment analysis, machine learning models, and LDA topic modeling to investigate the inter-city differences in EH, its underlying predictors, and the potential association with further climate change. Sentiment analysis revealed pronounced variations in EH across Chinese cities, with more positive emotions observed during weekends and holidays. Incorporating 17 potential predictors, we developed ten machine learning models. A random forest model achieved the best performance, with an R² that exceeded all other models by 1.05%–60.00% and an RMSE that was 7.41%–60.95% lower than the alternatives. SHAP analysis showed that landscape, socioeconomic, environmental, and geographic factors accounted for 24.58%–38.97%, 20.64%–40.12%, 11.96%–29.33%, and 11.47%–23.71% of the total feature importance in the EH prediction models, respectively. Among individual variables, the normalized difference vegetation index (NDVI) exhibited the highest feature importance, accounting for 18.56%–32.16% of the total importance, followed by per capita GDP, PM_2.5_ concentration, AQI, and temperature. Scenario-based projections suggest an association between projected climate warming and potential changes in urbanites’ EH. Overall, this study identifies the key predictors associated with urbanites’ EH and highlights the potential association of future climate warming with EH, providing valuable evidence for urban planning and policy interventions.

## Introduction

Urban development is shaped by the dynamic interplay between environmental conditions and human activities, which is reflected in mobility patterns and land use [1]. Beyond the physical landscape, this interplay also manifests in publicly expressed happiness (EH), representing people’s collective attitudes shaped by their thoughts and feelings, which serves as a vital social indicator for urban governance and planning [2–4]. In China, despite an average annual GDP growth of 8%, self-reported life satisfaction has not increased accordingly [5–7]. Such a disconnect highlights considerable spatial disparities in urbanites’ EH, potentially hindering development and affecting sectors such as tourism and the economy [8]. Therefore, a comprehensive understanding of this spatial heterogeneity and its driving factors is essential for promoting inclusive and sustainable urban development.

Urbanites’ EH, often measured through self-reported happiness, is a widely used metric in evaluating public goods and urban development [9,10]. However, traditional methods such as life satisfaction surveys are limited by small sample sizes, high costs, and their inability to capture real-time emotions [8,11–13]. Large-scale and long-term surveys using these methods across broad geographic areas remain difficult. In response, researchers increasingly rely on social media data, which provide real-time emotional expressions from diverse populations [14,15]. Social media has become a crucial data source for exploring public reactions to social and economic changes [16,17]. These big data sources are particularly valuable in mental health and urban studies, especially when combined with natural language processing (NLP) techniques that analyze emotional content in text [18]. Emotions expressed online can influence broader social dynamics, making sentiment analysis a powerful tool for assessing subjective well-being and offering new insights into EH and development [8,15,19–21].

Identifying the key predictors of urbanites’ EH is essential for effective city branding and tourism strategies [22]. Despite increasing attention to the diverse predictors for EH, existing studies tend to analyze these aspects separately, thus overlooking their combined and interactive effects. For example, although economic growth tends to improve overall happiness, its effects differ significantly across cities and income groups [23]. Extreme temperature is negatively related to EH, with cold weather having a stronger impact than heat [18]. Cheng et al. [24] reported that residents living near greener urban spaces, as measured by higher NDVI values, tend to be happier. Previous studies have reported associations between EH and a range of socioeconomic, geographic, landscape, and environmental factors [8,15,25,26]. However, most studies have focused on a limited set of associated factors, hindering a comprehensive understanding of their relative contributions to spatial variations in EH.

Climate change is among the most pressing challenges of the 21st century, with the Intergovernmental Panel on Climate Change (IPCC) projecting temperature increases of 1–5 °C and precipitation changes of 0–13% by 2100 [27–30]. Previous studies have demonstrated that climatic factors are significantly associated with EH [15,18,19]. For instance, Cheng et al. [19] found that the average city temperature is strongly associated with residents’ EH. Despite the well-established link between climate and emotional expression, and the likelihood of continued future climate warming, it remains unclear how such future climate change will affect urban EH patterns. This uncertainty is further complicated by the nonlinear and multi-factor nature of emotional dynamics under changing climatic conditions. In this context, machine learning provides a robust and cost-effective approach for projecting future EH responses by capturing complex interactions among multiple influencing variables [31,32]. It has been successfully applied to forecast urban development patterns and identify key influencing factors [33]. Leveraging these capabilities, machine learning can help predict how EH may shift under future climate scenarios, offering vital insights for adaptive planning and policymaking

The main objective of this study is as follows: (1) to examine the inter-city disparities of EH among urban residents across 50 Chinese cities; (2) to identify the key predictors underlying this uneven EH using social media data and machine learning algorithms; and (3) to explore the association between EH and future climate change. Notably, this study focuses on city-level structural factors associated with EH, while individual-level characteristics, which may also be related to variations in EH, are beyond the scope of the present analysis. Specifically, we analyze 5118772 geotagged Weibo posts from 50 cities using a SnowNLP-based sentiment analysis model to quantify city-level EH. To predict its inter-city spatial variation, we construct 10 machine learning models combined with LDA modeling. The best-performing model is subsequently used to identify the most influential predictors among 17 variables from socioeconomic, geographic, landscape, and environmental aspects. Finally, we assess the association between EH and future climate change by integrating future climate data into the machine learning framework. This study makes three key contributions:(1) revealing substantial inter-city disparities in urbanites’ EH across Chinese cities, providing an empirical basis for prioritizing cities with lower EH for targeted interventions; (2) identifying the most influential predictors of urban EH using machine learning models, offering actionable insights for enhancing urban residents’ happiness; (3) assessing the potential association of future climate change with urban EH, emphasizing residents’ emotional vulnerability to projected warming. Overall, this study offers novel insights into the dynamics of urban well-being and provides valuable guidance for adaptive urban planning and evidence-based policymaking in the context of environmental change.

## Literature review

### Analysis of expressed happiness through geotagged social media data

Despite Chinese sustained GDP growth over the past decades, the overall happiness of urban residents has not increased correspondingly [5, 6]. Self-reported happiness has risen far less than expected, reflecting pressures from inadequate public services, soaring housing costs, environmental degradation, and persistent concerns over food safety [8]. Traditional survey-based approaches and controlled studies of EH are limited in their ability to capture the immediate and dynamic emotional responses of large populations [11–13]. Happiness, sentiment, emotion, and well-being are related but distinct concepts. Emotion refers to short-term affective responses, sentiment reflects aggregated emotional expressions derived from text, happiness represents a broader evaluation of life satisfaction and positive affect, whereas well-being encompasses a wider range of emotional, cognitive, social, and psychological dimensions [18,22,24]. In this study, we focus on EH derived from social media, which captures population-level affective expression rather than well-being or happiness outcomes.

With the rise of the internet and the increasing interconnectedness among individuals, social media platforms such as Twitter and Weibo now allow researchers to monitor both individual and collective emotions continuously and at scale [34]. By tracking daily posts from users and analyzing their sentiment, it is possible to estimate the overall EH of urban residents in a given city [14,15]. Sina Weibo, one of China’s leading social media platforms with over 256 million daily active users and high levels of engagement [20], provides a particularly suitable channel for studying public attention and sentiment toward specific topics, such as green buildings [35], extreme temperatures [11–14,16,18], or urban parks [24]. For example, Wang et al. [18] collected 497,736 posts from 49 major Chinese cities to investigate the effects of extreme temperatures on EH, while other studies have analyzed public attention and sentiment toward green buildings and assessed the impact of urban parks on residents’ EH [35].

### The predictors of urbanites’ expressed happiness

Understanding the predictors behind EH is crucial for uncovering spatial heterogeneity in residents’ emotional expressions and for designing targeted policies to enhance urban governance and well-being [36,37]. Previous studies have identified a range of interrelated predictors for EH, including socioeconomic [19,25], geographic [15,18,36,38], environmental [8], and landscape [24,26,36] characteristics. For example, Cheng et al. [19] found that climatic and economic backgrounds largely determine disparities in urbanites’ EH during summer heat. Air pollution, indicated by PM2.5 concentration or the Air Quality Index, are significantly related to EH among Chinese urban residents [8], while access to urban parks with higher NDVI values is associated with increased EH [24]. Although long-term spatial differences in public sentiment are correlated with urban environmental features, these factors alone cannot fully explain the observed heterogeneity [3]. Moreover, as the number of potential predictors increases, traditional approaches are limited in ranking their relative importance and capturing complex interactions, leaving the specific contribution of each factor to EH unclear. This underscores the need for more advanced analytical techniques.

In recent years, machine learning has gained substantial attention in urban research due to its clear advantages over conventional statistical methods [33]. These approaches offer flexible frameworks for handling high-dimensional data, modeling nonlinear effects, and capturing complex interactions among predictors without strict parametric assumptions [32,39–41]. When combined with the SHAP method, machine learning models can quantify the contribution of each feature to predictions, enabling a clear understanding of the relative importance of multiple factors simultaneously [42]. Despite these methodological advances, no previous study has applied machine learning to investigate EH, even though it has the potential to explicitly determine the relative importance of various predictors to urban residents’ EH.

### Association between urbanites’ expressed happiness and future climate change

Global climate change is increasingly recognized as one of the most pressing challenges of the 21st century, with growing evidence indicating its substantial impact on psychological well-being [43]. Evidence from previous studies shows that air temperature significantly influences EH [11,18,19,36,38]. Projections by the IPCC indicate that global surface temperatures are expected to rise by 1–5 °C in the coming decades [27–30]. However, to date, no studies have systematically examined the association of future climate change with EH, leaving a critical knowledge gap. Understanding this relationship is of great importance, as it can provide insights into how urban populations may respond emotionally to warming trends, inform policies aimed at mitigating climate-related stressors, and guide urban planning strategies to enhance residents’ well-being under changing climatic conditions.

Empirical studies have demonstrated the sensitivity of urban residents’ EH to climatic conditions. For instance, perceived temperature thresholds for thermal discomfort are generally lower than official heat warning standards, and apparent temperature thresholds exhibit considerable variability across cities. Wang et al. [18] reported that extremely high or low temperatures are related to the decrease sentiment by approximately 0.161 and 0.272 units, respectively. Moreover, various weather conditions, including cold and hot temperatures as well as precipitation, are consistently associated with worsened sentiment expressions, even when weather-related posts are excluded [15]. Given the established link between temperature and emotional expression [11,18,19], these projected warming trends are likely related to the urban sentiment patterns, potentially exacerbating both spatial and temporal heterogeneity in EH. However, to date, no studies have systematically examined the association of future climate change with urban EH, highlighting a significant knowledge gap.

### Research gaps and our contributions

**Fig 1:**
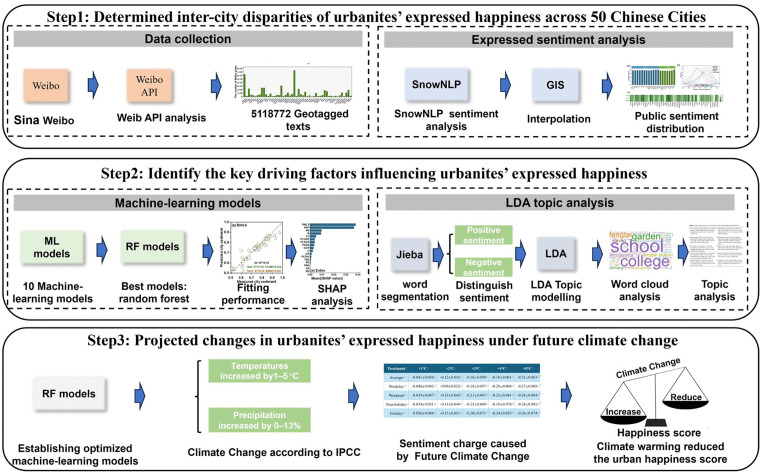
Research framework.

Overall, the research framework of this study is shown in Fig 1 and addresses two major research gaps in the investigation of EH. First, although previous studies have identified various socioeconomic, geographic, environmental, and landscape predictors for EH, they typically examine only a limited number of variables and do not assess their relative importance. To our knowledge, this study is the first to apply machine learning techniques to evaluate the association of 17 potential predictors with EH, and quantifying their relative contributions. Second, while prior research has demonstrated that climatic factors such as temperature and precipitation are significantly related to EH, the associations of future climate change with EH remain unclear. Using our machine learning framework, we predict how projected warming and increased precipitation scenarios associated with urban EH, providing new insights into the resilience and vulnerability of residents’ emotional well-being under changing climatic conditions. By integrating high-dimensional data analysis with climate projections, this study advances our understanding of the complex predictors of EH and offers practical guidance for urban planning and policy interventions aimed at enhancing residents’ well-being.

## Materials and methods

### Study area

Investigating EH in China is particularly important due to the country’s rapid urbanization, growing environmental pressures, and the widespread influence of digital platforms in shaping emotional expression [8]. China is geographically vast and hosts numerous cities, which makes it impractical to collect geotagged Weibo posts for all locations. To capture the most representative urban areas, we adopt the “2024 Top Chinese Cities Ranking” (http://www.warton.com.cn/Uploads/ueditor/file/20240731/66a99dd7ac785.pdf), which evaluates cities using both hard economic indicators (e.g., GDP, fiscal revenue) and soft indicators (e.g., environmental quality, education, healthcare, and culture). The top 50 cities (Table S1 in S1 File) are considered the most influential in Chinese economic and social development. These cities typically have larger populations, higher social media activity, and more stable volumes of geotagged posts, which provides a representative and sufficient dataset for examining urban emotional patterns.

### Geotagged social media data collection and processing

Social media platforms have emerged as powerful tools for capturing EH, offering broad coverage and low-cost alternatives compared to traditional methods such as questionnaires and mental maps [14,15]. In the era of big data, individuals are increasingly willing to share their thoughts and emotions online, making social media a rich resource for sentiment analysis [22]. Since its launch in 2009, Weibo has become China’s leading microblogging platform, widely used for sharing personal experiences and opinions. Geotagged texts on social media have gained popularity in evaluating urban sentiment, as they allow for spatially explicit assessments of public attitudes. Unlike passive data sources, Weibo posts actively reflect users’ perceptions, making them suitable for analyzing emotional responses to urban environments. Prior studies have used Weibo data to examine the evolution of public opinion, emotional perception, and attitudes toward urban issues [11,20,34,35].

Compared with other big data sources, such as mobile positioning data, geotagged social media texts are uniquely positioned to capture both the subjective emotions and the spatial context of city life. Checking in on social media is typically an intentional act, often reflecting moments that users find worth sharing. Given Twitter’s inaccessibility in China, Weibo has filled the gap as the country’s dominant microblogging platform, with over 256 million daily active users [20]. In this study, we collected Weibo posts from mainland China between January 1 and December 30, 2024, using a web crawler connected to the platform’s streaming API, in compliance with Weibo’ s terms of service. We retained only those posts with geotags, resulting in a dataset of 5118772 posts across 50 cities. Each record included information such as timestamp, post content, gender, user location, and geolocation coordinates. All geotagged Weibo posts used in this study contained only publicly accessible information. To protect participants’ privacy, all data, including textual content and check-in information, anonymized prior to analysis by removing usernames, profile links, and any other metadata that could potentially serve as personal identifiers. No personal identification information was collected, stored, or utilized at any stage of the research.

### Topic modelling

Latent Dirichlet Allocation (LDA) is a topic model that identifies the underlying topics of each document in a corpus through probabilistic distributions [44]. In this study, it is used to characterize the major discussion topics in the social media corpus. LDA is a three-layer Bayesian probabilistic model that includes the document layer, the topic layer, and the word layer. LDA clusters words into topics by analyzing the co-occurrence relationships between words, generating “document–topic” and “topic–word” distribution matrices. Due to its simplicity and wide applicability, LDA has been extensively used for analyzing topics in user reviews and for identifying emerging trends or popular topics [45].

The model’s performance was evaluated using perplexity and log-likelihood scores, calculated as follows:


$$\begin{array}{c} Perplexity=\text{exp}(-\frac{\text{1}}{N}\sum_{i=\text{1}}^{N}logP\left(w_{i}|\theta \right)) \end{array}$$

$$\begin{array}{c} Log-Likelihood=\sum_{d=\text{1}}^{D}\sum_{w=\text{1}}^{N_{d}}logp\left(W_{d}|\theta \right) \end{array}$$

Where $P\left(w_{i}|\theta \right)$ is the predicted probability of word $w_{i}$ in the model, *N* is the total number of words across all documents, $W_{d}$ refers to the words in document d, and $p\left(W_{d}|\theta \right)$ is the generation probability of word $W_{d}$ in document d. A lower perplexity and higher log-likelihood indicate a better model fit. After experimentation, we chose 14 topics to ensure each group contained a sufficient number of Weibo posts. The LDA model was implemented using the pyLDAvis package in Python (version 3.12.3), following word segmentation with the jieba package. Prior to conducting the LDA analysis, all words were translated into English using the googletrans package, after which the resulting topic outputs were manually reviewed to correct any mistranslations or semantically inconsistent keywords, ensuring the accuracy of the topic modeling. Word clouds were generated using the wordcloud package.

### Urbanites’ expressed happiness analysis

EH is determined using sentiment analysis, which is also known as opinion mining or polarity analysis and involves the process of analyzing subjective texts that express emotional content. It entails extracting underlying emotional tendencies and classifying the sentiment conveyed [35,46]. As a key research focus in fields such as Natural Language Processing (NLP), data mining, and web mining, sentiment analysis utilizes machine learning techniques to analyze and categorize the emotional tone of textual data. Its primary goal is to identify emotional tendencies and viewpoints in relation to entities and their attributes, providing valuable insights for public opinion analysis, user profiling, and recommendation systems.

Because emotions can diffuse through social networks and shape broader collective moods, sentiment analysis on social media has become increasingly important for understanding public well-being [16,17]. The integration of social media “big data” with NLP techniques enables the real-time extraction of emotional information from textual content [18–21]. Various sentiment analysis tools have been employed to quantify emotions in social media data, including SnowNLP [47,48] and Tencent’ s NLP [18]. SnowNLP, a Chinese NLP library based on TextBlob, is widely used for tasks such as Chinese word segmentation, sentiment analysis, and text classification [47,48]. It includes a built-in sentiment-labeled corpus for detecting sentiment in Chinese and employs a Naive Bayes classifier for training and prediction. SnowNLP has been widely adopted and validated in large-scale Chinese social media research due to its efficiency, interpretability, and ability to process massive volumes of user-generated content [47,49].

SnowNLP classifies text into positive and negative categories, with scores approaching 1 indicating positive sentiment and scores approaching 0 indicating negative sentiment. To improve data quality, we excluded posts likely to be advertisements, containing insufficient Chinese text, or exhibiting highly ambiguous linguistic cues such as sarcasm, exaggeration, or irony. A detailed description of the data cleaning procedure is provided in the Supplementary Information (SI). For each remaining post, only Chinese characters, excluding numbers, English letters, punctuation, URLs, hashtags, and mentions, were extracted to reduce noise commonly present in social media text during word segmentation. This approach is implemented using the SnowNLP package in Python (3.12.3), providing a simple and effective method for conducting sentiment analysis on Chinese texts.

### Machine-learning fitting of urbanites’ expressed happiness

#### Selection of predictive variables.

EH is influenced by a variety of predictors [3,15,22]. In this study, these variables were categorized into four major groups: socioeconomic, geographic, landscape, and environmental aspects. Socioeconomic aspects include population density (PD), Gross Domestic Product (GDP), per capita Gross Domestic Product (PCGDP), per capita consumption expenditure (PCCE), and per capita disposable income of urban residents (PCDIU), Average house price (AHP), all of which may be linked to sentiment expression in cities [19,22]. Geographic factors encompass climatic and topographic variables such as precipitation (P), air temperature (AT), humidity (H), and average altitude (AV). Lastly, environmental quality is represented by indicators such as the Air Quality Index (AQI) and PM2.5 concentration (PM2.5), both of which directly affect residents’ well-being and emotional expression [8]. Landscape factors include Normalized Difference Vegetation Index (NDVI), urbanization rate (UR), urban tourist area density (UTAD), Road density (RD), and City area (CA). The mean EH scores for each city, along with separate averages for weekdays, weekends, holidays, and non-holidays, were used as the dependent variable in the machine learning models. Pearson correlation analysis was conducted with a cutoff threshold of 0.85 to remove variables carrying redundant information [42].

#### Model development and performance evaluation.

To ensure comparability among input variables, data were standardized during model training using the StandardScaler from the scikit-learn Python package. 10 machine learning models were initially selected for predicting EH scores: Classification and Regression Trees (CART), Extremely Randomized Trees (ET), K-nearest neighbors (KNN), neural network models (NN), Support Vector Regression (SVR), Gradient Boosting Regression Tree (GBRT), Random Forest (RF), eXtreme Gradient Boosting (XGBoost), boosted regression tree (BRT), and Stochastic gradient descent regressor (SGDRegressor). Among these models, Random Forest showed the most promising performance, leading to its selection as the primary model for predicting average EH at the city level. Hyperparameters for each model were optimized using grid search to identify the best configuration, with the Random Forest model ultimately chosen for subsequent analysis. All machine learning methods were implemented using the scikit-learn package in Python (3.12.3).

#### Model validation and interpretation.

To assess model reliability and reduce overfitting, 5-fold cross-validation was applied to the models [39,41]. Model performance was evaluated using the coefficient of determination (R^2^), root-mean-square error (RMSE), mean square error (MSE), and mean absolute error (MAE) [40], calculated via the sklearn.metrics package in Python. Higher R^2^ values and lower RMSE, MAE, and MSE values indicate better predictive performance. The formulas used to compute these metrics are as follows:


$$\begin{array}{c} {\text{R}}^{\text{2}}=\text{1}-\frac{\sum_{\text{i}}{\left({\hat{\text{y}}}_{\text{i}}-{\text{y}}_{\text{i}}\right)}^{\text{2}}}{\sum_{\text{i}}{\left({\text{y}}_{\text{i}}-\overset{―}{\text{y}}\right)}^{\text{2}}} \end{array}$$

$$\begin{array}{c} \text{MSE}=\frac{\text{1}}{\text{n}}\sum_{\text{i}=\text{1}}^{\text{n}}{\left({\text{y}}_{\text{i}}-{\hat{\text{y}}}_{\text{i}}\right)}^{\text{2}} \end{array}$$

$$\begin{array}{c} \text{MAE}=\frac{\text{1}}{\text{n}}\sum_{\text{i}=\text{1}}^{\text{n}}\left|({\text{y}}_{\text{i}}-{\hat{\text{y}}}_{\text{i}})\right| \end{array}$$

$$\begin{array}{c} \text{RMSE}=\sqrt{\frac{\text{1}}{\text{n}}\sum_{\text{i}=\text{1}}^{\text{n}}{\left({\text{y}}_{\text{i}}-{\hat{\text{y}}}_{\text{i}}\right)}^{\text{2}}} \end{array}$$

where n is the samples number; $y_{i}$ represents the true value; ${\hat{y}}_{i}$ is the predicted value; and $\overset{―}{y}$ refers to the average value.

The Shapley Additive Explanations (SHAP) method, based on cooperative game theory, was used to quantify the relative contributions of different factors to EH. Each feature was assigned a SHAP value reflecting its individual impact on model output, and the mean absolute SHAP (MAS) value was used to summarize the overall importance of all descriptors [41]. The mean absolute SHAP value (MAS) was employed to quantify the overall contribution of each descriptor to the model output using test data, which reflects the model’s generalizable patterns.

### Prediction of urbanites’ expressed happiness under future climate change

Global climate change is recognized as one of the most critical challenges of the 21st century. According to the IPCC, global temperatures are projected to rise by 1–5 °C, and precipitation is expected to change by 0–13% by the end of the century [27–30]. To explore how such future climatic shifts, specifically warming and altered precipitation, may be associated with urban emotional expression, we applied an optimized predictive model. Following previous studies [50], we simulated changes in air temperature (1–5 °C) and precipitation (0–13%) for each city. The potential relationship of climate change on EH were then assessed by comparing the differences in sentiment values between current and projected future climatic conditions.

## Results

### Topics of geotagged social media

**Fig 2:**
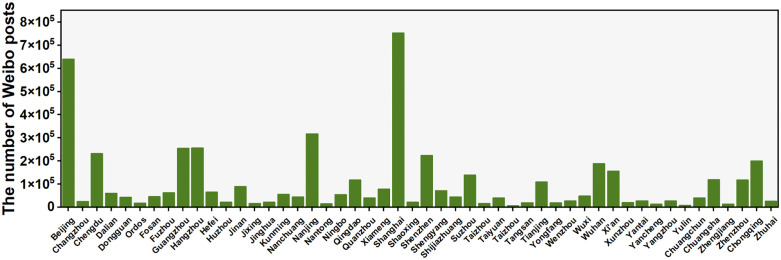
The number of Weibo posts across Chinese cities.

The spatial distribution of 5118772 Weibo posts across 50 Chinese cities is illustrated in Fig 2. The number of Weibo posts collected from different cities varied substantially, ranging from 7,907–752,599. Among the sampled cities, Beijing and Shanghai had the highest number of posts, with 640,938 and 752,599 entries, respectively, whereas Taizhou and Yulin had the lowest counts, at 7,907 and 8,617 posts.

**Fig 3:**
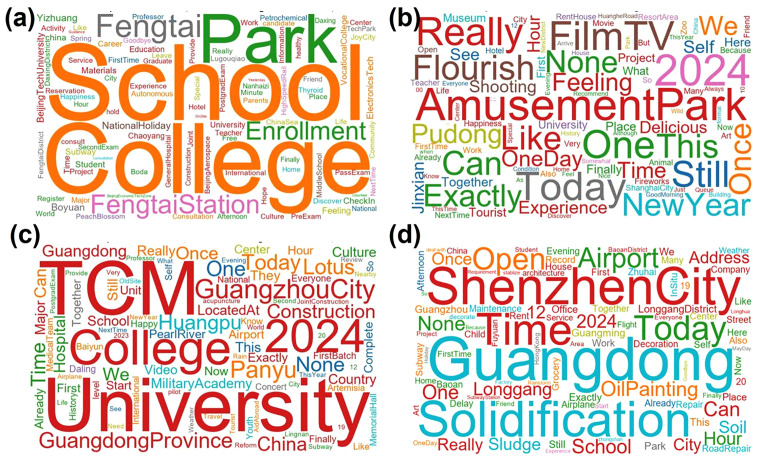
The word cloud map of representative Chinese cities including Beijing (a), Shanghai (b), Guangzhou (c), Shenzhen (d).

To uncover the latent themes embedded in public discourse, we employed the LDA model, which extracts topics based on word co-occurrence patterns [51]. This probabilistic approach enables the identification of underlying public concerns that are not directly observable from surface-level text. As shown in Fig S1 in S1 File and Fig 3a–d, word-cloud representations highlight topic distributions across the country and in four representative cities. Table S2-S3 in S1 File summarizes the primary themes and representative keywords extracted from textual data across all 50 cities. The LDA clustering results demonstrate clear semantic separability and robust topic coherence. Fourteen prominent thematic clusters were identified, covering a wide range of domains such as tourism, culture, leisure, social interaction, and employment, and whether. The identified topic words, such as parks, weather, holidays, temperature, and work, reflect recurring themes in public discourse that are likely to influence EH.

### Spatiotemporal distribution of urbanites’ expressed happiness across 50 Chinese cities

**Fig 4:**
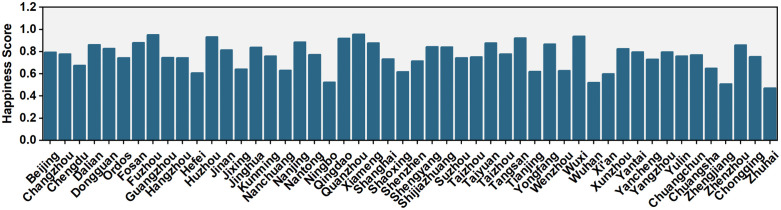
The average EH score across 50 Chinese cities.

The distribution of EH is illustrated in Fig 4, revealing significant inter-city variation. Cities of Zhuhai, Zhenjiang, and Wuhan reported the most negative average sentiment scores, at 0.47, 0.51, and 0.52, respectively. In contrast, cities including Quanzhou, Fuzhou, and Wuxi exhibited the most positive sentiment, reaching 0.96, 0.95, and 0.94 (Fig 4). Notably, Chinese four first-tier cities including Beijing, Shanghai, Guangzhou, and Shenzhen, with the score of 0.79, 0.73, 0.75 and 0.72, did not rank among the most positive score. These findings underscore the substantial heterogeneity in urban emotional expression across cities, highlighting the need for a deeper investigation into the underlying predictors.

**Fig 5:**
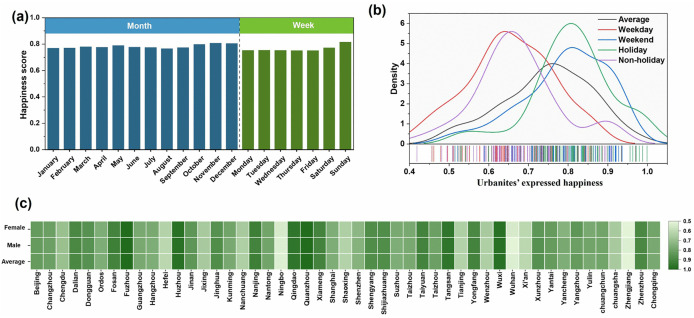
(a) Average EH score in different month and weeks (a). Distribution curves of EH score in weekday, weekend, non-holiday, and holiday (b). Average happiness score in different gender across 50 Chinese Cities (c).

We further examined how EH varies across demographic groups and time periods. As shown in Fig 5, gender differences in EH were minimal, with variations remaining within 5%. Happiness scores across different months were also relatively stable, ranging from 0.77 to 0.81. In contrast, noticeable weekly fluctuations were observed: happiness values on weekends were significantly more positive than those on weekdays. Specifically, Saturday and Sunday recorded more positive scores of 0.78 and 0.86, respectively, compared to 0.71–0.74 from Monday to Friday. EH became substantially more positive during holidays, increasing from approximately 0.65 on non-holiday days to around 0.83 during holidays.

### Key predictors for urbanites’ expressed happiness

**Fig 6:**
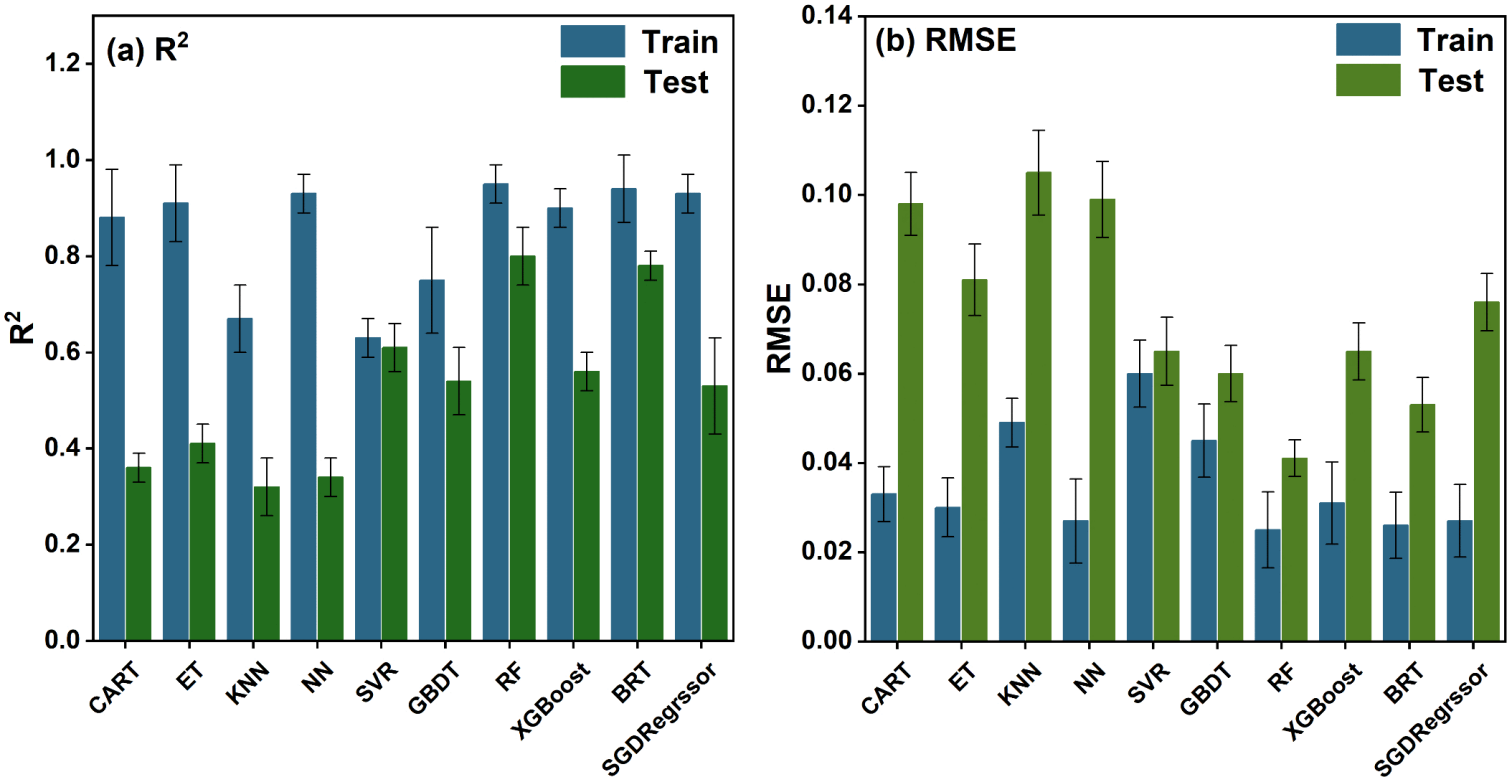
The fitting R^2^ and RSME value of 10 machine-learning models. CART: Classification and Regression Trees; ET: Extremely Randomized Trees; KNN: K-nearest neighbors; NN: neural network models; SVR: Support Vector Regression; GBRT: Gradient Boosting Regression Tree; RF: Random Forest; XGBoost: eXtreme Gradient Boosting; BRT: boosted regression tree, and SGDRegressor: Stochastic gradient descent regressor.

To explore the influencing factors behind EH, we selected 17 representative variables encompassing socioeconomic, geographic, landscape, and environmental dimensions. 10 machine learning models were developed to predict the average EH across cities. Among them, the Random Forest model demonstrated the best predictive performance, achieving the highest R^2^ and the lowest RMSE, MSE, and MAE values (Fig 6, Table S9 in S1 File), with an R^2^ that exceeded all other models by 1.05%–60.00% and an RMSE that was 7.41%–60.95% lower than the alternatives. Given that EH is also influenced by temporal factors such as holidays and weekdays, the Random Forest model was further applied to predict happiness scores under five temporal scenarios, including average, non-holiday, holiday, weekday, and weekend scores.

**Fig 7:**
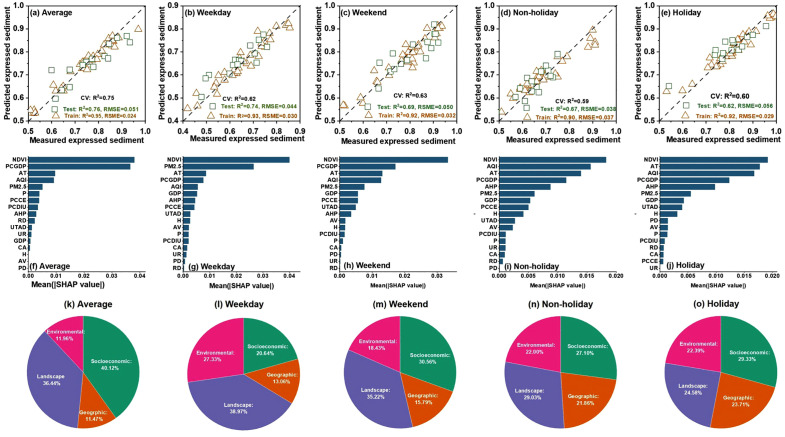
Prediction performance of random-forest models in EH (a-e). Corresponding analyses of the respective explanatory power of each model variable (f-j). PD: population density, GDP: Gross Domestic Product, PCGDP: per capita Gross Domestic Product, PCCE: per capita consumption expenditure, PCDIU: per capita disposable income of urban residents, AHP: average house price; P: precipitation, AT: air temperature, H: humidity, AV: average altitude, AQI: air quality index, and PM2.5: PM2.5 concentration, NDVI: Normalized Difference Vegetation Index, UR: urbanization rate, UTAD: urban tourist area density, RD: road density, and CA: city area.

The RF model demonstrated strong performance, with R^2^ values exceeding 0.62 and 0.90 on the testing and validation sets, respectively, and an average cross-validated R^2^ above 0.59 (Fig 7a–7e). These results indicate good predictive accuracy and no signs of overfitting. To further interpret the model, the relative contributions of individual predictors were quantified using the SHAP method [42,52]. Specifically, SHAP values were employed to assess the relative importance of the 17 input variables, which were grouped into four categories: socioeconomic, geographic, environmental, and landscape aspects. Among all predictors, the NDVI, a landscape factor, consistently ranked as the most influential predictors across all data groups, associated with 18.56%−32.16% of total models importance (Fig 7f–7o). Other key predictors included PCGDP from the socioeconomic group, PM2.5 and AQI from the environmental group, and AT from the geographic group. Quantitatively, the relative importance of each predictors category to EH predictive models ranged as follows: socioeconomic factors (20.64%–40.12%), geographic factors (11.47%–23.71%), environmental factors (11.96%–29.33%), and landscape factors (24.58%–38.97%). Overall, these findings suggest that EH is simultaneously related to multiple urban attributes, with NDVI, PCGDP, PM2.5, AQI, and AT identified as the dominant predictors.

### Climate–happiness associations under future climate change

Global climate change is widely acknowledged as one of the most pressing challenges of the 21st century, with growing evidence indicating its impact on human psychological well-being. Based on our simulation results, a 1 °C, 2 °C, 3 °C, 4 °C, and 5 °C increase in temperature could be associated with a decrease in EH by approximately 0.036–0.041, 0.09–0.15, 0.15–0.21, 0.18–0.24, and 0.21–0.28 points, respectively (Table 1). These values correspond to an average reduction of 4.4–41.2% with a 1 °C increase in temperature, indicating a strong association between EH and warming conditions (Table S8 in S1 File). This trend suggests an association between future climate warming and reductions in urbanites’ EH. In contrast, projected increases in precipitation (ranging from 1% to 13%) showed only a weak association with urban EH, with estimated changes remaining within ±0.1 points. Overall, these findings underscore the potential psychological risks of future climate warming for urban populations and emphasize the need for adaptive urban planning and mental health considerations in climate resilience strategies.

**Table 1:** Climate–happiness associations under future climate warming and increased rainfall.

Treatment	+1°C	+2°C	+3°C	+4°C	+5°C
Average	−0.041 ± 0.036	−0.12 ± 0.042	−0.16 ± 0.059	−0.18 ± 0.061	−0.21 ± 0.063
Weekday	−0.046 ± 0.042	−0.09 ± 0.052	−0.18 ± 0.057	−0.20 ± 0.064	−0.27 ± 0.068
Weekend	−0.039 ± 0.047	−0.13 ± 0.045	−0.21 ± 0.047	−0.22 ± 0.041	−0.28 ± 0.084
Non-holiday	−0.034 ± 0.031	−0.11 ± 0.049	−0.15 ± 0.069	−0.19 ± 0.078	−0.24 ± 0.091
Holiday	−0.036 ± 0.044	−0.15 ± 0.051	−0.20 ± 0.071	−0.24 ± 0.055	−0.26 ± 0.074
**Treatment**	**+1% mm**	**+4% mm**	**+7% mm**	**+10% mm**	**+13% mm**
Average	+0.013 ± 0.042	−0.026 ± 0.052	−0.054 ± 0.062	−0.084 ± 0.054	−0.097 ± 0.041
Weekday	−0.014 ± 0.064	−0.023 ± 0.041	−0.059 ± 0.052	−0.091 ± 0.051	−0.063 ± 0.058
Weekend	+0.018 ± 0.040	−0.017 ± 0.039	−0.064 ± 0.052	−0.072 ± 0.037	−0.078 ± 0.049
Non-holiday	+0.014 ± 0.041	−0.024 ± 0.043	−0.057 ± 0.057	−0.093 ± 0.032	−0.087 ± 0.054
Holiday	+0.0091 ± 0.039	−0.017 ± 0.047	−0.055 ± 0.048	−0. 04 ± 0.039	−0.068 ± 0.045

## Discussion

### Uneven spatial distribution on urbanites’ expressed happiness

EH has emerged as a critical social indicator for assessing the effectiveness of urban governance and planning [8,53,54]. Fluctuations in EH not only reflect residents’ quality of life but also affect key sectors such as tourism and service-related activities [55]. Social media, as a powerful tool for capturing real-time, large-scale emotional expressions, provides valuable insights into EH and urban dynamics, especially when combined with NLP techniques [56–59].

Our findings highlight significant spatial heterogeneity in EH across Chinese cities, revealing that economic prosperity alone may not ensure more positive emotional satisfaction (Fig 4). Notably, first-tier cities such as Beijing, Shanghai, Guangzhou, and Shenzhen do not rank among the happiest, suggesting that predictors beyond economic development also play a crucial role. Consistently, SHAP analysis shows that urban EH is not only associated with socioeconomic indicators but also with environmental factors, including NDVI, PM_2.5_ concentration, AQI, and temperature. Socioeconomic factors contribute less than 30% to the model importance, highlighting that no consistent pattern is observed between economic levels and EH values across cities.

Understanding the temporal and demographic patterns of EH is crucial for developing inclusive and responsive urban policies that address the emotional needs of diverse populations. However, research on these patterns remains limited. We found minimal gender differences and a stable emotional climate across months, consistent with previous studies showing little gender divergence in aggregated mood expressions on online platforms (Fig 5a, 5c). Prior studies also found limited gender divergence in aggregated mood expression in online platforms [22]. Zhang et al. [33] found that weekly-related fluctuations significantly affect urban leisure segregation index.

### The key predictors for urbanites’ expressed happiness

Machine learning models reveal that RF model demonstrated the best predictive performance, with an R^2^ that exceeded all other models by 1.05%–60.00% and an RMSE that was 7.41%–60.95% lower than the alternatives (Table S9 in S1 File). The strong performance of the RF model demonstrates the practical value of our framework. It captures complex non-linear and interacting effects more effectively than traditional models, enabling a more reliable identification of key influencing factors. Its stable predictive accuracy also allows cities to anticipate changes in residents’ emotional well-being under different conditions, including future climate scenarios. Moreover, SHAP interpretability clarifies both the importance of each predictors, providing actionable guidance for targeted urban interventions.

EH is related to a complex interplay of socioeconomic, geographic, environmental, and landscape factors. These four categories were associated with happiness variation by approximately 20.64%–40.12%, 11.47%–23.71%, 11.96%–29.33%, and 24.58%–38.97%, respectively (Fig 7). This finding contrasts with previous studies, which often considered only a narrow set of factors and therefore yielded limited insights into the drivers of urban sentiment [3,8,36]. In contrast, our results highlight that EH emerges from the combined association with multiple interdependent predictors rather than any single dimension. This integrative approach provides a more comprehensive understanding of emotional dynamics in urban spaces, offering a solid foundation for designing multidimensional policies and developing sentiment-sensitive urban planning strategies.

Among all predictors, the NDVI exhibited the highest feature importance in the EH prediction models, accounting for approximately 18.56% to 32.16% of the total importance (Fig 7). This finding is consistent with previous studies by He et al. [22,36], who demonstrated a significant relationship between NDVI and urban emotional expression. In addition to NDVI, several other key predictors were also identified. PCGDP, one of socioeconomic factors, showed a substantial association on EH. Cheng et al. [19] found that economic background significantly determined disparities in urban residents’ EH during summer heatwaves. Environmental factors such as PM2.5 and the AQI also played important roles. Air pollution has been shown to negatively affect health [60,61], cognitive performance [62], labour productivity [63] and later-life educational outcomes [64], all of which may indirectly diminish emotional expression. Empirical evidence from Zheng et al. [8] further confirmed that air pollution indicators, including PM2.5 and AQI, were negatively correlated with expressed happiness on social media. Geographic factors, particularly AT, were also related to EH variation. Numerous studies have consistently found a significant relationship between temperature and EH [11,18,19,36]. Taken together, these findings highlight that urban emotional expression is related to a multifaceted set of interrelated preditors. Recognizing the relative importance of each predictor offers valuable insights for designing holistic, emotionally responsive urban policies.

### Climate–EH associations under future climate warming

Global climate change is increasingly recognized as a critical challenge of the 21st century, with mounting evidence suggesting its significant on psychological well-being [43]. Existing literature, along with our findings, consistently demonstrates that air temperature plays a crucial ssociations with EH [11,18,19,36]. According to projections by the IPCC, global surface temperatures are expected to increase by 1–5 °C in the coming decades [27–30]. Given the established relationship between temperature and emotional expression, such climatic shifts are likely related to change in urban EH. However, to date, no studies have systematically examined the association of future climate change with urban EH, leaving a significant gap in the literature.

Based on our machine learning model evaluation, we arrived at an insightful conclusion: there exists a strong negative correlation between rising temperatures and EH. Specifically, a 1 °C, 2 °C, 3 °C, 4 °C, and 5 °C increase in temperature is associated with an approximate decrease in EH by 0.036–0.041, 0.09–0.15, 0.15–0.21, 0.18–0.24, and 0.21–0.28 points, respectively (Table 1, Table S8 in S1 File). These results indicate that as global warming continues, cities may experience a gradual deterioration in residents’ emotional well-being. The negative emotional response to heat may be linked to physical discomfort, reduced outdoor activity, and increased irritability, as highlighted in previous psychological and behavioral studies. Moreover, the cumulative effect of other heat-related stressors, such as poor air quality and urban heat island intensification, may further exacerbate the decline in public sentiment. These findings underscore the urgent need for climate-adaptive urban planning strategies that prioritize thermal comfort, green infrastructure, and resilience in mental health.

The analysis assumes time-invariant relationships between climatic variables and EH, which is a necessary simplification for identifying EH sensitivity to projected climatic changes. However, this assumption does not capture adaptive processes at individual and urban levels, where behavioral, technological, and infrastructural changes may reshape these relationships under climate change. Therefore, the results should be interpreted as scenario-based estimates under current climate–emotion relationships rather than deterministic forecasts of future emotional well-being. This limitation highlights the need for future research incorporating dynamic adaptation processes.

## Conclusions

This study quantified the spatial heterogeneity of EH and identified its key predictors using 5118772 geotagged Weibo posts from 50 Chinese cities. EH was notably more positive during weekends and holidays. SHAP analysis showed that landscape, socioeconomic, environmental, and geographic factors accounted for 24.58%–38.97%, 20.64%–40.12%, 11.96%–29.33%, and 11.47%–23.71% of the total feature importance in the EH prediction models, respectively. Among individual variables, the NDVI exhibited the highest feature importance, accounting for 18.56%–32.16% of the total importance. Future climate simulations suggest that climate warming is associated with change in urban EH, while precipitation changes show minimal associations with EH. These findings highlight the emotional sensitivity of urban residents to climate warming and provide actionable recommendations for urban policymakers.

Despite the use of a large dataset and comprehensive analyses, this study has several limitations. First, it relies on geotagged Weibo posts from 50 Chinese cities, which may limit the generalizability of findings across different cultural and regional contexts. Second, as an observational study, causal relationships between predictors and expressed happiness cannot be established. Third, projections under future climate scenarios depend on IPCC models and assumptions, and actual EH outcomes may vary due to inherent uncertainties. Forth, although SnowNLP showed satisfactory validation performance for large-scale social media analysis, it may not fully capture complex contextual semantics and nuanced emotional expressions. Future studies could incorporate deep-learning approaches and large language models to further improve sentiment measurement accuracy. Finally, given the observational nature of our data and the machine-learning framework employed, the findings should be interpreted as statistical associations rather than causal effects. Future studies could build on this work by employing quasi-experimental designs, longitudinal analyses, or instrumental variable approaches to better explore potential causal relationships.

## Supporting information

S1 File Supporting Text S1-S3, Figure S1 and Tables S1–S13. (DOCX)
