# Supplementary material for: Unraveling uneven urbanites’ expressed happiness across Chinese cities using geotagged social media data: Key predictors and future climate–happiness associations
Source: PLoS One. 2026 Jul 16;21(7):e0353996. doi: 10.1371/journal.pone.0353996 (PMC13374925; doi:10.1371/journal.pone.0353996)
Supplement: S1 File — (DOCX) [file pone.0353996.s001.docx]

**Supplementary Material for**

**Unraveling uneven urbanites’ expressed happiness across Chinese cities using geotagged social media data: key predictors and future climate–happiness associations**

Yibiao Li ^a^, Hui Zhong ^c^, Yufei Dong ^d^, Lei Lu ^b, *^

^a^ School of Arts and Design, Lianyungang Technical College, 222000 Lianyungang, China

^b^ College of Environment & Ecology, Hunan Agricultural University, 410127 Changsha, China

^c^ Design Department, Shanghai Normal University, 201815 Shanghai, China

^d^ Arts Visual Communication Design Department, Shandong University of Arts, 250300 Shandong, China

*** Corresponding Author:**

**Lei Lu** (Dr.): E-mail: LeiLu0127@163.com

**ORCID:** https://orcid.org/0000-0003-1390-6410

**TEL Phone:**  +86 191 9820 4380

**Supplementary Text S1. Data collection of 17 predictors**

Socioeconomic variables, including population density (PD), Gross Domestic Product (GDP), per capita Gross Domestic Product (PCGDP), per capita consumption expenditure (PCCE), per capita disposable income of urban residents (PCDIU), urbanization rate (UR), and city area (CA), were obtained from the China City Statistical Yearbook 2024 and the statistical bulletins of individual cities published by the National Bureau of Statistics of China. Population density was calculated as the ratio of the resident population to the administrative area of each city. Average house price (AHP) data were collected from the China Real Estate Price Platform and supplemented by official city statistical reports when necessary.

Climatic variables, including precipitation (P), air temperature (AT), and relative humidity (H), were obtained from the China Meteorological Data Service Center. Average altitude (AV) was derived from the Shuttle Radar Topography Mission (SRTM) Digital Elevation Model (DEM) dataset. Environmental quality indicators, including the Air Quality Index (AQI) and PM2.5 concentration (PM2.5), were obtained from the China National Environmental Monitoring Center and the Tracking Air Pollution in China (TAP) dataset, respectively.

Landscape-related variables were derived from multiple geospatial datasets. The Normalized Difference Vegetation Index (NDVI) was calculated using MODIS satellite imagery accessed through Google Earth Engine. Road density (RD) was computed based on road network data obtained from OpenStreetMap.

**Supplementary Text S2. Manual validation of the SnowNLP method**

Given that the objective of this study is to investigate city-level variations in expressed happiness rather than develop sentiment analysis algorithms, the use of the well-established SnowNLP framework is appropriate and consistent with previous research [1, 2]. To evaluate its performance, we conducted a manual validation by randomly selecting 100 posts on three times, and comparing independent human annotations with SnowNLP classifications. The agreement rates were 89%, 85%, and 91%, respectively, yielding an average accuracy of 88.3%. These results indicate a high level of consistency between manual evaluation and SnowNLP outputs, supporting the reliability of the framework for large-scale analyses of urban expressed happiness.

**Supplementary Text S3. Weibo Data Preprocessing and Cleaning**

To improve data quality and ensure that the dataset primarily reflected individual users’ emotional expressions, several preprocessing procedures were implemented. First, posts associated with accounts lacking user nicknames were removed, as such records could not be reliably attributed to individual users. Second, reposted posts were excluded by identifying repost indicators (e.g., “转发微博” and “//@”). Third, institutional and organizational accounts were removed using a keyword-based screening strategy applied to account names and profile descriptions. Fourth, advertising and promotional content was identified and excluded based on marketing-related keywords, as well as explicit commercial solicitation, product promotion, and purchasing information. Detailed keyword lists used for account and content filtering are provided in Table S10.

To reduce linguistic ambiguity, posts containing sarcastic or exaggerated expressions were excluded using a rule-based filtering strategy, including: (i) sarcasm-related lexicons (e.g., “真棒”, “呵呵”, “笑死”, “你真行啊”); (ii) repeated punctuation (e.g., “!!!”, “???”, “……”); and (iii) character elongation patterns (e.g., “哈哈哈哈”, “好好好好好”). In addition, posts containing emojis with ambiguous or mixed emotional valence (e.g., 😏 🙃 😅 🤔 😶 😐 😑 😬) were removed to further reduce sentiment uncertainty. Finally, to enhance the sentiment lexicon and capture emerging online expressions, we incorporated widely used Chinese internet buzzwords based on the “Top 30 Chinese Internet Buzzwords of 2024” compiled by Sohu (https://www.sohu.com/a/840483367_121846117).

Nevertheless, fully eliminating all forms of noise from large-scale social media datasets remains challenging. Some residual institutional, promotional, automated, duplicated, near-duplicated, and potentially bot-generated content may still exist, which could introduce additional uncertainty into sentiment estimates. Future studies could incorporate more advanced account-classification, text-similarity detection, and bot-identification techniques to further improve data quality and enhance the robustness of city-level sentiment analyses.

**Table S1** The selection of 50 Chines cities

| City name | | | | |
| --- | --- | --- | --- | --- |
| Beijing | Hefei | Qingdao | Taiyuan | Yantai |
| Changzhou | Huzhou | Quanzhou | Taizhou | Yancheng |
| Chengdu | Jinan | Xiameng | Tangsan | Yangzhou |
| Dalian | Jixing | Shanghai | Tianjing | Yulin |
| Dongguan | Jinghua | Shaoxing | Yongfang | Chuangchun |
| Ordos | Kunming | Shenzhen | Wenzhou | Chuangsha |
| Fosan | Nanchuang | Shengyang | Wuxi | Zhengjiang |
| Fuzhou | Nanjing | Shijiazhuang | Wuhan | Zhenzhou |
| Guangzhou | Nantong | Suzhou | Xi'an | Chongqing |
| Hangzhou | Ningbo | Taizhou | Xunzhou | Zhuhai |

**Table S2** Examples of topics 1-7 in the result of LDA topic modelling

|  | Topic 1 | Topic 2 | Topic 3 | Topic 4 | Topic 5 | Topic 6 | Topic 7 |
| --- | --- | --- | --- | --- | --- | --- | --- |
| 1 | Zoo | Shenyang | Really | Museum | today | one | ancient town |
| 2 | Panda | History | Fuzhou | Dalian | still | not have | Xiamen |
| 3 | Taizhou | Wuxi | Taiyuan | Garden | concert | city | here |
| 4 | Jinan | Ningbo | Quanzhou | Weather | Yantai | like | Nanjing |
| 5 | May Day | Erdos | Concert | Shijiazhuang | Xuzhou | hospital | Chengdu |
| 6 | Also | Located | Oneself | Yancheng City | Nanchang | exactly | Suzhou |
| 7 | Animal | Video | Also | Nature | together | Nantong | Chongqing |
| 8 | Vacation | Travel | World | Feel | tourist | the people | tourist |
| 9 | Tourist | Culture | Experience | Building | Foshan | can | together |
| 10 | Work | China | Together | May Day | one | work | also |
| 11 | Video | Tourist | One | History | also | I | China |
| 12 | Not have | The people | City | City | we | Travel | one |
| 13 | Experience | Also | China | Vacation | time | located in | history |
| 14 | One | Experience | Exactly | China | China | time | located in |
| 15 | Travel | World | Culture | together | Zhenjiang | building | feel |
| 16 | We | Nature | Like | experience | experience | together | can |
| 17 | exactly | feel | Taizhou | world | exactly | career | time |
| 18 | like | one | we | time | city | also | city |
| 19 | I | can | not have | tourist | 2024 | here | building |
| 20 | can | May Day | 2024 | animal | can | world | experience |

**Table S3** Examples of topics 8-14 in the result of LDA topic modelling

|  | Topic 8 | Topic 9 | Topic 10 | Topic 11 | Topic 12 | Topic 13 | Topic 14 |
| --- | --- | --- | --- | --- | --- | --- | --- |
| 1 | Beijing | career | park | Shenzhen | 2024 | activity | we |
| 2 | Jiaxing | university | hotspot | Guangzhou | Shanghai | Tianjin | this one |
| 3 | nunja | college | Hefei | Zhuhai | first | toray | can |
| 4 | Foshan | Hangzhou | campus | Taizhou | Wuhan | Yulin | Changsha |
| 5 | Dongguang | school | grand sight | Guangdong | Shaoxing | library | Huzhou |
| 6 | Caspian Sea | Changchun | world | together | Zhenjiang | work | Zhengzhou |
| 7 | nature | Wenzhou | QINGDAO | the people | China | Travel | one |
| 8 | Tianjin | Wuxi | Tangshan | Zhengzhou | time | time | experience |
| 9 | one | Suzhou | college | park | world | experience | together |
| 10 | feel | together | time | building | also | May Day | feel |
| 11 | experience | first | animal | world | city | culture | time |
| 12 | located in | work | vacation | Foshan | the people | vacation | also |
| 13 | China | China | tourist | Dongguang | located in | feel | here |
| 14 | Tangshan | city | together | I | concert | first | the people |
| 15 | Travel | time | experience | two thousand and twenty-four | experience | China | first |
| 16 | tourist | campus | can | time | together | together | still |
| 17 | world | culture | work | like | one | the people | tourist |
| 18 | here | can | China | today | feel | building | Travel |
| 19 | together | located in | located in | first | animal | tourist | like |
| 20 | can | 2024 | Travel | Temperature | history | also | culture |

**Table S4** The variance of key performance metrics across cross-validation folds, including R², RMSE, MAE, and MSE

| Index | Set | Average | Weekday | Weekend | Non-holiday | Holiday |
| --- | --- | --- | --- | --- | --- | --- |
| R^2^ | Test | 0.82±0.12 | 0.78±0.14 | 0.76±0.13 | 0.74±0.11 | 0.75±0.18 |
|  | Tain | 0.95±0.0084 | 0.93±0.010 | 0.92±0.011 | 0.90±0.009 | 0.92±0.013 |
| MAE | Test | 0.033±0.0133 | 0.035±0.015 | 0.031±0.014 | 0.036±0.017 | 0.038±0.017 |
|  | Tain | 0.017±0.0019 | 0.019±0.0021 | 0.021±0.0019 | 0.026±0.0027 | 0.022±0.0023 |
| MSE | Test | 0.0019±0.0011 | 0.0021±0.0013 | 0.0023±0.0014 | 0.0026±0.0015 | 0.0024±0.0016 |
|  | Tain | 0.0005±0.0001 | 0.0006±0.0001 | 0.0007±0.0001 | 0.0006±0.0001 | 0.0006±0.0002 |
| RMSE | Test | 0.042±0.0144 | 0.048±0.016 | 0.046±0.015 | 0.049±0.011 | 0.048±0.014 |
|  | Tain | 0.022±0.0019 | 0.025±0.0021 | 0.027±0.0023 | 0.028±0.0021 | 0.031±0.0028 |

**Table S5** The main functions and hyperparameters of 10 machine learning models.

| Model | gamma | C | leaf_size | n_neighbors | alpha | max_ depth | max_leaf _nodes | n_estimators | min_child _weight |  | activation | learning_rate |
| --- | --- | --- | --- | --- | --- | --- | --- | --- | --- | --- | --- | --- |
| CART |  |  |  |  |  | 3 | 6 |  |  |  |  |  |
| ET |  |  |  |  |  | 3 | 6 |  |  |  |  |  |
| KNN |  |  | 14 | 5 |  |  |  |  |  |  |  |  |
| NN |  |  |  |  |  |  |  |  |  |  | relu | 0.001 |
| SVR | 0.1 | 1 |  |  |  |  |  |  |  |  |  |  |
| GBDT |  |  |  |  |  | 3 |  | 42 |  |  |  |  |
| RF |  |  |  |  |  | 10 |  | 25 |  |  |  |  |
| XGBoost |  |  |  |  |  |  |  | 24 | 3 |  |  |  |
| BRT |  |  |  |  |  | 3 |  | 29 |  |  |  |  |
| SGDRegressor |  |  |  |  | 0.001 |  |  |  |  |  |  |  |

**Table S6** The 95% confidence interval (95% CI) of the predicted EH under future climate warming and increased rainfall

| Treatment | +1℃ | +2℃ | +3℃ | +4℃ | +5℃ |
| --- | --- | --- | --- | --- | --- |
| Average | [-0.067, -0.015] | [-0.151, -0.089] | [-0.201, -0.119] | [-0.224, -0.136] | [-0.247, -0.173] |
| Weekday | [-0.073, -0.019] | [-0.124, -0.056] | [-0.212, -0.148] | [-0.226, -0.174] | [-0.292, -0.248] |
| Weekend | [-0.074, -0.004] | [-0.149, -0.111] | [-0.231, -0.189] | [-0.237, -0.203] | [-0.314, -0.246] |
| Non-holiday | [-0.049, -0.019] | [-0.159, -0.061] | [-0.226, -0.074] | [-0.245, -0.135] | [-0.315, -0.165] |
| Holiday | [-0.063, -0.009] | [-0.170, -0.130] | [-0.229, -0.171] | [-0.266, -0.214] | [-0.287, -0.233] |
| Treatment | +1% mm | +4% mm | +7% mm | +10% mm | +13% mm |
| Average | [-0.056, 0.082] | [-0.070, 0.018] | [-0.086, -0.022] | [-0.114, -0.054] | [-0.065, -0.129] |
| Weekday | [-0.160, 0.132] | [-0.073, 0.027] | [-0.072, -0.046] | [-0.108, -0.074] | [-0.096, -0.030] |
| Weekend | [-0.034, 0.070] | [-0.030, -0.004] | [-0.077, -0.051] | [-0.085, -0.059] | [-0.089, -0.067] |
| Non-holiday | [-0.041, 0.069] | [-0.056, 0.008] | [-0.083, -0.031] | [-0.097, -0.089] | [-0.111, -0.063] |
| Holiday | [-0.042, 0.060] | [-0.056, 0.022] | [-0.084, -0.026] | [-0.060, -0.016] | [-0.081, -0.055] |

**Table S7** The calculated Mean SHAP value and its associated standard deviation (STD) and 95% CI.

|  | Mean | STD | 95% CI |
| --- | --- | --- | --- |
| PD | 2.53E-06 | 9.69E-08 | [2.46E-06, 2.60E-06] |
| AV | 3.57E-06 | 1.72E-07 | [3.44E-06, 3.69E-06] |
| H | 3.71E-05 | 1.60E-06 | [3.59E-05, 3.82E-05] |
| CA | 5.02E-04 | 2.29E-05 | [4.86E-04, 5.18E-04] |
| GDP | 7.29E-04 | 2.91E-05 | [7.08E-04, 7.50E-04] |
| UR | 1.05E-03 | 6.07E-05 | [1.00E-03, 1.09E-03] |
| UTAD | 1.53E-03 | 7.16E-04 | [1.01E-03, 2.04E-03] |
| RD | 2.20E-03 | 6.30E-04 | [1.74E-03, 2.65E-03] |
| AHP | 2.80E-03 | 4.63E-04 | [2.47E-03, 3.13E-03] |
| PCDIU | 3.37E-03 | 5.86E-04 | [2.95E-03, 3.79E-03] |
| PCCE | 3.93E-03 | 1.95E-04 | [3.79E-03, 4.07E-03] |
| P | 3.94E-03 | 5.08E-04 | [3.58E-03, 4.30E-03] |
| PM2.5 | 5.32E-03 | 5.40E-04 | [4.93E-03, 5.70E-03] |
| AQI | 8.95E-03 | 7.21E-04 | [8.43E-03, 9.46E-03] |
| AT | 9.29E-03 | 3.31E-03 | [6.92E-03, 1.17E-02] |
| PCGDP | 3.12E-02 | 3.71E-03 | [2.85E-02, 3.38E-02] |
| NDVI | 3.72E-02 | 5.59E-03 | [3.32E-02, 4.12E-02] |

**Table S8** The decreased percentage of EH under future climate warming

| Treatment | Average | Weekday | Weekend | Non-holiday | Holiday |
| --- | --- | --- | --- | --- | --- |
| +1℃ | 5.5% | 7.0% | 4.9% | 5.1% | 4.4% |
| +2℃ | 16.1% | 13.7% | 16.4% | 16.4% | 18.5% |
| +3℃ | 21.4% | 27.5% | 26.5% | 22.3% | 24.7% |
| +4℃ | 24.1% | 30.5% | 27.8% | 28.3% | 29.7% |
| +5℃ | 28.1% | 41.2% | 35.4% | 35.7% | 32.1% |

**Table S9** The prediction performance of 10 learning models in the train, test and cross-validation R^2^ for urbanites’ expressed happiness.

| Model | Train | | | | Test | | | | cross-validation R^2^ |
| --- | --- | --- | --- | --- | --- | --- | --- | --- | --- |
|  | R^2^ | RMSE | MSE | MAE | R^2^ | RMSE | MSE | MAE |  |
| Classification and Regression Trees (CART) | 0.880 | 0.033 | 0.001 | 0.024 | 0.360 | 0.098 | 0.010 | 0.075 | 0.510 |
| Extremely randomized trees (ET) | 0.910 | 0.030 | 0.001 | 0.020 | 0.410 | 0.081 | 0.007 | 0.068 | 0.320 |
| K-nearest neighbors (KNN) | 0.670 | 0.049 | 0.002 | 0.046 | 0.320 | 0.105 | 0.011 | 0.080 | 0.410 |
| neural network models (NN) | 0.930 | 0.027 | 0.001 | 0.017 | 0.340 | 0.099 | 0.010 | 0.076 | 0.250 |
| Support vector regression (SVR) | 0.630 | 0.060 | 0.004 | 0.050 | 0.610 | 0.065 | 0.004 | 0.052 | 0.280 |
| Gradient boosting decision tree (GBDT) | 0.750 | 0.045 | 0.003 | 0.034 | 0.540 | 0.060 | 0.004 | 0.044 | 0.630 |
| Random forest (RF) | 0.950 | 0.025 | 0.001 | 0.016 | 0.800 | 0.041 | 0.002 | 0.031 | 0.750 |
| Extreme gradient boosting (XGBoost) | 0.900 | 0.031 | 0.001 | 0.021 | 0.560 | 0.065 | 0.004 | 0.057 | 0.510 |
| boosted regression tree (BRT) | 0.940 | 0.026 | 0.001 | 0.018 | 0.780 | 0.053 | 0.003 | 0.041 | 0.630 |
| Stochastic gradient descent regressor (SGDRegressor) | 0.930 | 0.027 | 0.001 | 0.019 | 0.530 | 0.076 | 0.006 | 0.061 | 0.610 |

**Table S10** Keywords used in the data cleaning process

| Reposts posts | institutional and organizational accounts | advertising and promotional accounts |
| --- | --- | --- |
| 转发微博 (Repost Weibo Post), //@ (Forwarding Marker / Repost Marker) | 政府 (Government), 市委 (Municipal Party Committee), 人大 (People's Congress), 政协 (Chinese People's Political Consultative Conference, CPPCC), 委员会 (Committee), 局 (Bureau), 公安 (Public Security), 检察院 (People's Procuratorate), 法院 (Court), 税务 (Taxation), 应急管理 (Emergency Management), 生态环境 (Ecological Environment), 发布 (Official Release), 政务 (Government Affairs), 日报 (Daily News), 晚报 (Evening News), 晨报 (Morning News), 时报 (Times), 都市报 (Metropolitan News), 新闻网 (News Network), 新闻 (News), 传媒 (Media Group), 媒体 (Media), 电视台 (Television Station), 广播 (Broadcasting), 频道 (Channel), 客户端 (Client App), 观察 (Observer), 周刊 (Weekly Magazine), 网 (Web Portal), 大学 (University), 学院 (College), 研究院 (Research Institute), 研究所 (Research Institute), 实验室 (Laboratory), 附属医院 (Affiliated Hospital), 校友会 (Alumni Association), 招生 (Admissions), 继续教育 (Continuing Education), 医院 (Hospital), 卫生院 (Health Center), 疾控中心 (Center for Disease Control and Prevention, CDC), 妇幼保健院 (Maternal and Child Health Hospital), 医学中心 (Medical Center), 集团 (Group Corporation), 公司 (Company), 企业 (Enterprise), 银行 (Bank), 证券 (Securities), 保险 (Insurance), 基金 (Fund), 科技 (Technology), 地产 (Real Estate), 投资 (Investment), 控股 (Holding), 商城 (Shopping Mall), 店 (Store), 客服 (Customer Service) | 优惠 (Discount Offer), 促销 (Sales Promotion), 折扣 (Discount), 秒杀 (Flash Sale), 福利 (Benefits), 返现 (Cashback), 代购 (Purchasing Agent Service), 招商 (Business Recruitment), 加盟 (Franchise), 推广 (Promotion), 广告 (Advertisement), 客服 (Customer Service) |

**Table S11** Data cleaning results showing removal rates and remaining sample size of Weibo posts

| Step | Removal Percentage | Remaining Posts |
| --- | --- | --- |
| Initial dataset | – | 5,118,772 |
| Missing user nickname | 5.91% | 4,816,253 |
| Retweeted posts | 12.24% | 4,226,743 |
| Institutional accounts | 12.23% | 3,709,813 |
| Advertisement content | 4.31% | 3,549,920 |
| Ambiguous/contradictory posts | 1.20% | 3,507,321 |

**Table S12** The geographic and socioeconomic predictors of the selected 50 cities.

| Cities | Geographic factors | | | | Socioeconomic factors | | | | | |
| --- | --- | --- | --- | --- | --- | --- | --- | --- | --- | --- |
|  | AT (°C) | P (mm) | AV (m) | H (%) | PD (persons/km²) | GDP (10⁸ CNY) | PCGDP (CNY/person) | PCCE (CNY/person) | PCDIU (CNY/person) | AHP (CNY/m²) |
| Beijing | 14 | 631 | 455 | 0.6 | 7.5 | 62919.5 | 12.8 | 40897 | 81650 | 72263 |
| Changzhou | 15 | 1217 | 14 | 0.7 | 8.1 | 32749.6 | 21.0 | 41695 | 61744 | 15520 |
| Chengdu | 17 | 1045 | 913 | 0.8 | 6.7 | 30654.5 | 11.0 | 32171 | 54897 | 19750 |
| Dalian | 12 | 1007 | 27 | 0.7 | 16.7 | 22439.0 | 12.6 | 43023 | 61904 | 14133 |
| Dongguan | 24 | 1982 | 24 | 0.8 | 2.4 | 43187.9 | 8.7 | 30983 | 57286 | 27394 |
| Ordos | 7 | 347 | 1260 | 0.5 | 390.5 | 72777.0 | 18.7 | 40487 | 56000 | 7042 |
| Fosan | 22 | 1801 | 39 | 0.5 | 4.0 | 31302.8 | 13.9 | 34120 | 63000 | 16504 |
| Fuzhou | 19 | 1837 | 257 | 0.8 | 14.2 | 19133.9 | 16.8 | 37181 | 55638 | 20232 |
| Guangzhou | 21 | 1751 | 98 | 0.8 | 4.0 | 125618.7 | 16.5 | 29480 | 50501 | 46473 |
| Hangzhou | 16 | 1627 | 186 | 0.8 | 13.5 | 26947.7 | 17.5 | 35133 | 50587 | 42892 |
| Hefei | 15 | 1314 | 30 | 0.8 | 11.6 | 11458.7 | 13.7 | 32964 | 51609 | 20434 |
| Huzhou | 16 | 1582 | 65 | 0.8 | 34.8 | 237442.4 | 19.3 | 46788 | 74400 | 7250 |
| Jinan | 13 | 612 | 112 | 0.6 | 11.0 | 57213.6 | 14.3 | 37831 | 62506 | 17395 |
| Jixing | 18 | 1093 | 5 | 0.8 | 7.6 | 105259.9 | 13.6 | 47551 | 55909 | 18500 |
| Jinghua | 19 | 1300 | 316 | 0.8 | 22.0 | 65413.5 | 9.7 | 25061 | 73639 | 19248 |
| Kunming | 16 | 1033 | 1996 | 0.7 | 24.2 | 62291.5 | 9.5 | 39231 | 55501 | 13065 |
| Nanchuang | 20 | 1559 | 42 | 0.7 | 11.0 | 40774.2 | 11.9 | 32515 | 52622 | 12563 |
| Nanjing | 17 | 1277 | 28 | 0.7 | 6.9 | 25762.2 | 19.4 | 46552 | 79858 | 11364 |
| Nantong | 17 | 1314 | 4.5 | 0.8 | 10.3 | 81003.0 | 16.0 | 38897 | 62512 | 15868 |
| Ningbo | 18 | 1552 | 92 | 0.8 | 10.1 | 107323.2 | 8.6 | 27916 | 50144 | 25890 |
| Qingdao | 15 | 573 | 37 | 0.7 | 10.6 | 85528.9 | 16.1 | 59663 | 65751 | 10211 |
| Quanzhou | 22 | 1659 | 352 | 0.8 | 12.4 | 55865.5 | 14.7 | 37116 | 60697 | 10755 |
| Xiameng | 22 | 1503 | 29 | 0.8 | 3.0 | 49902.9 | 14.1 | 47411 | 52880 | 39247 |
| Shanghai | 18 | 1044 | 13 | 0.8 | 2.6 | 86765.8 | 11.7 | 18111 | 89477 | 71421 |
| Shaoxing | 19 | 1124 | 180 | 0.8 | 15.3 | 45950.4 | 13.8 | 49300 | 54392 | 19950 |
| Shenzhen | 23 | 1935 | 48 | 0.8 | 1.1 | 25875.0 | 11.7 | 21793 | 26910 | 68946 |
| Shengyang | 7 | 570 | 155 | 0.6 | 14.0 | 48440.7 | 9.9 | 40232 | 53650 | 10897 |
| Shijiazhuang | 12 | 517 | 337 | 0.6 | 14.1 | 65514.7 | 7.3 | 37956 | 47564 | 14127 |
| Suzhou | 18 | 1407 | 6 | 0.7 | 6.7 | 66352.8 | 15.6 | 50656 | 52989 | 23688 |
| Taizhou | 17 | 1842 | 141 | 0.8 | 15.1 | 29062.3 | 9.9 | 44180 | 53879 | 17687 |
| Taiyuan | 9 | 486 | 1195 | 0.6 | 13.2 | 69213.0 | 19.9 | 44948 | 65835 | 10289 |
| Taizhou | 15 | 1259 | 7 | 0.8 | 12.9 | 26855.2 | 15.6 | 39192 | 59604 | 11743 |
| Tangsan | 12 | 692 | 90 | 0.6 | 17.5 | 43653.2 | 13.0 | 39906 | 50000 | 10418 |
| Tianjing | 14 | 565 | 64 | 0.6 | 8.8 | 48048.1 | 13.2 | 31586 | 55355 | 26157 |
| Yongfang | 15 | 535 | 42 | 0.7 | 17.7 | 64515.5 | 18.8 | 59396 | 71155 | 11196 |
| Wenzhou | 19 | 1449 | 217 | 0.8 | 13.9 | 50979.5 | 13.0 | 23333 | 77973 | 19905 |
| Wuxi | 18 | 1266 | 8 | 0.7 | 6.2 | 79491.3 | 11.7 | 28409 | 76644 | 18086 |
| Wuhan | 18 | 1177 | 48 | 0.8 | 6.2 | 27785.0 | 7.7 | 26684 | 51693 | 18950 |
| Xi'an | 16 | 572 | 974 | 0.6 | 7.8 | 53661.7 | 7.2 | 27431 | 51178 | 17723 |
| Xunzhou | 16 | 955 | 50 | 0.7 | 13.0 | 33574.6 | 20.1 | 36937 | 44796 | 10336 |
| Yantai | 11 | 641 | 30 | 0.7 | 18.5 | 40171.0 | 15.3 | 45605 | 57126 | 8582 |
| Yancheng | 16 | 902 | 3 | 0.8 | 25.2 | 46327.8 | 16.3 | 26884 | 48526 | 11333 |
| Yangzhou | 15 | 1283 | 10 | 0.8 | 14.4 | 77449.3 | 17.0 | 35572 | 56781 | 14873 |
| Yulin | 9 | 439 | 1271 | 0.6 | 30.6 | 23842.0 | 14.9 | 45142 | 52000 | 10313 |
| chuangchun | 5 | 495 | 215 | 0.6 | 4.4 | 18825.5 | 18.4 | 52336 | 75480 | 8450 |
| chuangsha | 19 | 1445 | 180 | 0.8 | 11.2 | 56434.0 | 14.5 | 42936 | 57276 | 21471 |
| Zhengjiang | 17 | 1406 | 20 | 0.7 | 11.9 | 97491.1 | 6.2 | 22866 | 44602 | 21182 |
| Zhenzhou | 17 | 471 | 162 | 0.6 | 0.9 | 37270.7 | 17.2 | 48710 | 53000 | 14129 |
| Chongqing | 20 | 1036 | 754 | 0.7 | 25.8 | 84387.3 | 9.1 | 31531 | 47435 | 12529 |
| Zhuhai | 21 | 1919 | 22 | 0.8 | 6.9 | 45258.8 | 18.0 | 43526 | 67773 | 26151 |

**Table S13** The Environmental and landscape predictors of 50 selected cities

| Cities | Environmental factors | | Landscape factors | | | |
| --- | --- | --- | --- | --- | --- | --- |
|  | PM2.5 (μg/m³) | AQI | NDVI | UR (%) | RD (km/km²) | CA (km2) |
| Beijing | 32 | 0.79 | 17.5 | 87.8 | 1.83 | 79.2 |
| Changzhou | 35 | 0.78 | 8.1 | 78.5 | 1.92 | 76.7 |
| Chengdu | 39 | 0.68 | 6.7 | 80.5 | 1.95 | 78.1 |
| Dalian | 24 | 0.86 | 36.7 | 82.9 | 1.07 | 92.6 |
| Dongguan | 70 | 0.83 | 2.4 | 92.8 | 1.23 | 58.8 |
| Ordos | 19 | 0.74 | 29.5 | 79.2 | 0.97 | 88.0 |
| Fosan | 61 | 0.88 | 4.0 | 95.4 | 1.41 | 87.7 |
| Fuzhou | 52 | 0.95 | 11.2 | 73.3 | 0.97 | 98.1 |
| Guangzhou | 23 | 0.75 | 4.0 | 86.8 | 1.78 | 90.4 |
| Hangzhou | 36 | 0.74 | 13.5 | 84.2 | 0.44 | 84.4 |
| Hefei | 51 | 0.61 | 7.6 | 85.6 | 0.16 | 86.1 |
| Huzhou | 22 | 0.93 | 34.8 | 68.8 | 1.33 | 89.5 |
| Jinan | 38 | 0.81 | 11.0 | 75.3 | 0.79 | 66.7 |
| Jixing | 66 | 0.64 | 7.6 | 74.5 | 0.79 | 83.6 |
| Jinghua | 23 | 0.84 | 22.0 | 81.1 | 1.21 | 97.5 |
| Kunming | 72 | 0.76 | 24.2 | 82.3 | 1.09 | 99.7 |
| Nanchuang | 85 | 0.63 | 11.0 | 79.6 | 1.62 | 74.6 |
| Nanjing | 29 | 0.89 | 36.9 | 87.2 | 1.50 | 81.9 |
| Nantong | 27 | 0.77 | 10.3 | 72.6 | 2.19 | 83.6 |
| Ningbo | 76 | 0.52 | 6.1 | 79.9 | 0.17 | 53.5 |
| Qingdao | 28 | 0.92 | 20.6 | 78.3 | 1.78 | 96.7 |
| Quanzhou | 20 | 0.96 | 12.4 | 70.8 | 1.80 | 98.1 |
| Xiameng | 81 | 0.88 | 13.0 | 90.8 | 0.35 | 79.7 |
| Shanghai | 25 | 0.73 | 2.6 | 89.5 | 2.05 | 87.1 |
| Shaoxing | 66 | 0.62 | 5.3 | 73.1 | 0.58 | 96.4 |
| Shenzhen | 61 | 0.72 | 1.1 | 99.8 | 1.38 | 62.1 |
| Shengyang | 37 | 0.84 | 24.0 | 85.1 | 1.06 | 82.7 |
| Shijiazhuang | 46 | 0.84 | 24.1 | 72.3 | 1.25 | 74.1 |
| Suzhou | 61 | 0.74 | 6.7 | 82.5 | 0.31 | 83.6 |
| Taizhou | 84 | 0.75 | 15.1 | 66.7 | 1.32 | 93.4 |
| Taiyuan | 38 | 0.88 | 23.2 | 89.5 | 1.99 | 77.8 |
| Taizhou | 63 | 0.78 | 12.9 | 70.1 | 1.79 | 75.3 |
| Tangsan | 35 | 0.92 | 17.5 | 66.7 | 1.50 | 87.5 |
| Tianjing | 41 | 0.62 | 8.8 | 85.5 | 1.27 | 66.8 |
| Yongfang | 27 | 0.87 | 27.7 | 66.1 | 1.84 | 91.0 |
| Wenzhou | 34 | 0.63 | 13.9 | 74.7 | 1.29 | 97.5 |
| Wuxi | 32 | 0.94 | 6.2 | 83.3 | 0.89 | 82.5 |
| Wuhan | 75 | 0.52 | 6.2 | 84.8 | 0.58 | 59.2 |
| Xi'an | 52 | 0.6 | 6.8 | 79.9 | 0.59 | 82.1 |
| Xunzhou | 31 | 0.83 | 13.0 | 67.6 | 0.23 | 70.7 |
| Yantai | 27 | 0.8 | 28.5 | 69.2 | 1.53 | 88.8 |
| Yancheng | 32 | 0.73 | 25.2 | 64.9 | 1.49 | 83.4 |
| Yangzhou | 25 | 0.8 | 14.4 | 71.0 | 1.48 | 79.3 |
| Yulin | 29 | 0.76 | 20.6 | 63.5 | 2.93 | 97.7 |
| chuangchun | 24 | 0.77 | 24.4 | 68.2 | 1.03 | 99.0 |
| chuangsha | 68 | 0.65 | 7.2 | 83.6 | 0.38 | 87.9 |
| Zhengjiang | 56 | 0.51 | 5.9 | 80.7 | 0.80 | 54.5 |
| Zhenzhou | 29 | 0.86 | 22.9 | 79.4 | 1.20 | 71.9 |
| Chongqing | 51 | 0.76 | 25.8 | 71.7 | 0.05 | 91.0 |
| Zhuhai | 39 | 0.47 | 6.9 | 90.9 | 1.52 | 81.8 |

**
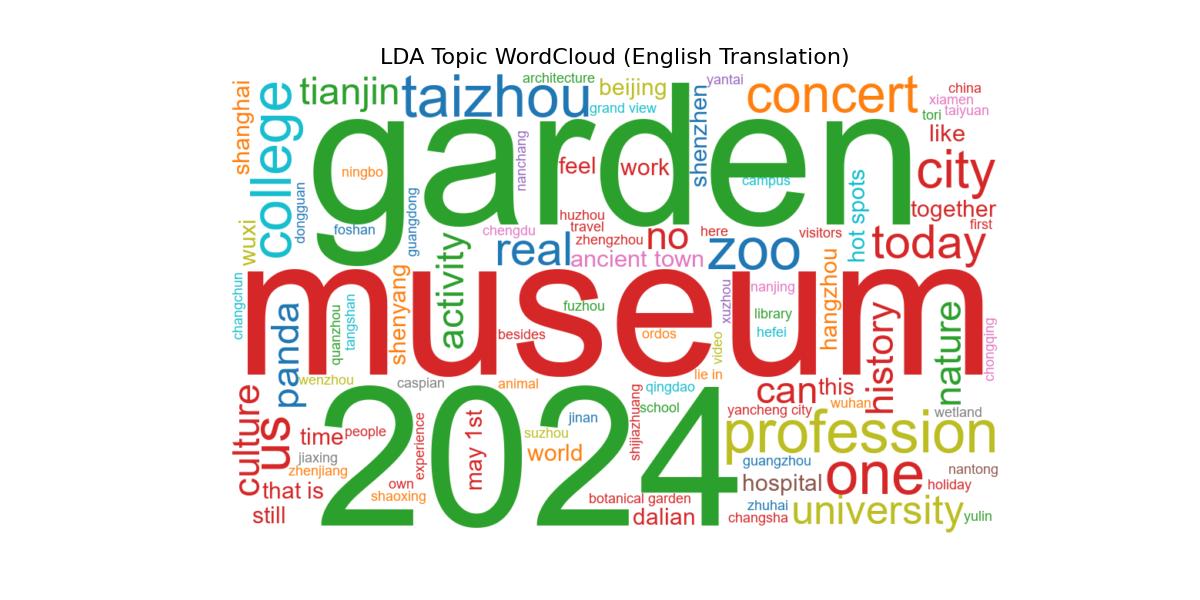
**

**Figure S1** The word cloud map using Weibo posts of total of 50 cities.

**Reference**

[1] M. Taitian, W. Yuhao, H. Wenjia, Content Mining and Sentiment Analysis of Online Comments for Ethnic Museums in Autonomous Regions, ECONOMIC GEOGRAPHY, 43 (2023) 230-236.

[2] C. Li, Y. Niu, L. Wang, How to win the green market? Exploring the satisfaction and sentiment of Chinese consumers based on text mining, Computers in Human Behavior, 148 (2023) 107890.
